# Construction and Comprehensive Analysis of Dysregulated Long Noncoding RNA-Associated Competing Endogenous RNA Network in Moyamoya Disease

**DOI:** 10.1155/2020/2018214

**Published:** 2020-06-13

**Authors:** Xuefeng Gu, Dongyang Jiang, Yue Yang, Peng Zhang, Guoqing Wan, Wangxian Gu, Junfeng Shi, Liying Jiang, Bing Chen, Yanjun Zheng, Dingsheng Liu, Sufen Guo, Changlian Lu

**Affiliations:** ^1^Research Department, Shanghai University of Medicine & Health Science Affiliated Zhoupu Hospital, Shanghai, China; ^2^Shanghai Key Laboratory of Molecular Imaging, Shanghai University of Medicine & Health Sciences, Shanghai, China; ^3^Department of Cardiology, Pan-Vascular Medicine Institute, Shanghai Tenth People's Hospital, Tongji University School of Medicine, Shanghai, China; ^4^Key Laboratory of Cancer Prevention and Treatment of Heilongjiang Province, Mudanjiang Medical University, Mudanjiang, China; ^5^Department of Pathology, Hongqi Hospital Affiliated to Mudanjiang Medical University, Mudanjiang, China; ^6^School of Clinical Medicine, Shanghai University of Medicine & Health Sciences, Shanghai, China; ^7^Department of Neurosurgery, Affiliated Hospital of Guangdong Medical University, Zhanjiang, Guangdong, China; ^8^Department of Oncology and Hematology, Shanghai University of Medicine & Health Sciences Affiliated Zhoupu Hospital, China

## Abstract

**Background:**

Moyamoya disease (MMD) is a rare cerebrovascular disease characterized by chronic progressive stenosis or occlusion of the bilateral internal carotid artery (ICA), the anterior cerebral artery (ACA), and the middle cerebral artery (MCA). MMD is secondary to the formation of an abnormal vascular network at the base of the skull. However, the etiology and pathogenesis of MMD remain poorly understood.

**Methods:**

A competing endogenous RNA (ceRNA) network was constructed by analyzing sample-matched messenger RNA (mRNA), long non-coding RNA (lncRNA), and microRNA (miRNA) expression profiles from MMD patients and control samples. Then, a protein-protein interaction (PPI) network was constructed to identify crucial genes associated with MMD. Gene Ontology (GO) and Kyoto Encyclopedia of Genes and Genomes pathway (KEGG) enrichment analyses were employed with the DAVID database to investigate the underlying functions of differentially expressed mRNAs (DEmRNAs) involved in the ceRNA network. CMap was used to identify potential small drug molecules.

**Results:**

A total of 94 miRNAs, 3649 lncRNAs, and 2294 mRNAs were differentially expressed between MMD patients and control samples. A synergistic ceRNA lncRNA-miRNA-mRNA regulatory network was constructed. Core regulatory miRNAs (miR-107 and miR-423-5p) and key mRNAs (STAT5B, FOSL2, CEBPB, and CXCL16) involved in the ceRNA network were identified. GO and KEGG analyses indicated that the DEmRNAs were involved in the regulation of the immune system and inflammation in MMD. Finally, two potential small molecule drugs, CAY-10415 and indirubin, were identified by CMap as candidate drugs for treating MMD.

**Conclusions:**

The present study used bioinformatics analysis of candidate RNAs to identify a series of clearly altered miRNAs, lncRNAs, and mRNAs involved in MMD. Furthermore, a ceRNA lncRNA-miRNA-mRNA regulatory network was constructed, which provides insights into the novel molecular pathogenesis of MMD, thus giving promising clues for clinical therapy.

## 1. Introduction

Moyamoya disease (MMD) is a rare cerebrovascular disease characterized by chronic progressive occlusion or stenosis of the bilateral internal carotid artery (ICA), the anterior cerebral artery (ACA), and the middle cerebral artery (MCA) [1, 2]. MMD is secondary to the formation of an abnormal vascular network at the base of the skull. Because the abnormal vascular network of the skull base looks like “smoke” on cerebral angiography images, it is called “moyamoya disease” [3]. The MMD incidence rate in Eastern Asian countries is higher [4], and it mainly occurs in children and young adults, peaking at the ages of 5 to 9 and 35 to 45 years [5]. MMD can seriously affect the mental and physical health of patients. However, the etiology and pathogenesis of MMD remain poorly understood; it may be related to genetics, inflammation, immune response, and environmental factors [6–11].

Many studies have reported that the ring finger protein 213 (RNF213) gene is an important susceptibility gene for MMD in East Asia, especially the p.R4810K variant [12–17]. However, MMD also occurs in patients without mutations in RNF213. To date, new candidate risk-MMD genes, such as the vascular smooth muscle cell-specific isoform of *α*-actin (ACTA2) [18, 19], endothelial nitric oxide synthase (eNOSase) [20], soluble guanylyl cyclase alpha subunit (GUCY1A3) [21], matrix metalloproteinases (MMPs) [22–26], tissue inhibitor of metalloproteinases (TIMPs) [23, 24], transforming growth factor *β*1 (TGF-*β*1) [27], Sortilin 1 (SORT1) [28], Connexin 43 (Cx43) [29], and caveolin-1 (Cav-1) [30, 31], have been continuously reported to be associated with MMD.

Moreover, with the development of microarray and sequencing technology, investigators have begun to explore factors other than direct disease-causing genes, including noncoding RNAs (ncRNAs). Gao et al. revealed the expression profile of lncRNAs and mRNAs in MMD patients in 2016 [9], and Dai et al. analyzed miRNAs in the serum of MMD patients and healthy controls in 2012 [32]. miRNAs can posttranscriptionally regulate gene expression by binding to MREs (miRNA-response elements) of their target transcript. mRNAs, lncRNAs, and other RNA transcripts could act as endogenous miRNA sponges to inhibit miRNA function. These interactions illustrate the famous ceRNA hypothesis presented by Salmena in 2011 [33], which gave us a new “language” in different types of RNA transcripts. After that, the ceRNA hypothesis was applied to many fields [34]. The Linc2GO database was constructed by Liu et al. in 2013 [35]. StarBase v2.0 was published by Li et al. to predict miRNA-ceRNA interactions [36]. Moreover, continued analysis of ceRNA networks would deepen our knowledge about how different subtypes of noncoding RNAs work with each other.

In this study, a comprehensive analysis of the miRNA, mRNA, and lncRNA expression profiles in MMD was done, and then, we constructed MMD-specific ceRNA networks using a large cohort from an online database. As far as we know, this is the first study to establish a ceRNA lncRNA-miRNA-mRNA network in MMD, which provides novel insight into the molecular pathogenesis of MMD, thus giving promising clues for clinical therapy. In addition, core regulatory miRNAs (miR-107 and miR-423-5p) and key mRNAs (STAT5B, FOSL2, CEBPB, and CXCL16) were enriched in immune system/inflammation biological processes, indicating their potential role in MMD.

## 2. Materials and Methods

### 2.1. Data Collection

miRNA microarray data were downloaded from Gene Expression Omnibus (GEO, https://www.ncbi.nlm.nih.gov/geo/) in NCBI (The National Center for Biotechnology Information). GEO is an unrestricted open access repository that provides high-throughput microarray and next-generation sequence datasets that have been submitted by researchers around the world. GSE45737 is a miRNA expression profile of the serum from 10 MMD patients and 10 normal healthy controls [32]. The lncRNA and mRNA expression profiles in blood samples from 15 MMD patients and 10 healthy controls were kindly provided by a collaborating academician, Zhao [9].

### 2.2. Identification of Differentially Expressed RNAs in MMD Patients Compared to Healthy Controls

R software with packages ggplot2, edgeR, and pheatmap (http://bioconductor.org/bioclite. R) was adopted to identify differentially expressed RNAs (DERs). In brief, datasets were standardized after conversion of formats, variance normalization, and the addition of missing values as well as statistical testing of differentially expressed probes. The expression levels of all targets, including mRNA, miRNA, and lncRNA, within the datasets were subjected to analysis with R. The threshold was set as a *P* value < 0.05 and ∣log_2_FC | >1. According to these criteria, DERs were identified for further analysis.

### 2.3. Gene Ontology and Pathway Enrichment Analyses

The Database of Annotation, Visualization and Integrated Discovery (DAVID, http://david.ncifcrf.gov) is a public database with comprehensive online tools for functional annotation. Kyoto Encyclopedia of Genes and Genomes (KEGG) is a collection of databases that contain information about genomes, biological pathways, diseases, and chemical substances [37]. Gene Ontology (GO) is an international standardized gene functional classification system that offers a dynamically updated controlled vocabulary and a strictly defined concept to comprehensively describe properties of genes and their products in any organism. GO has three ontologies: molecular function, cellular component, and biological process [38].

In the present study, GO and KEGG pathway enrichment analyses were performed using DAVID. *P* < 0.05 was considered statistically significant.

### 2.4. Construction of the ceRNA (lncRNA-miRNA-mRNA) Regulatory Network

**Figure 1:**
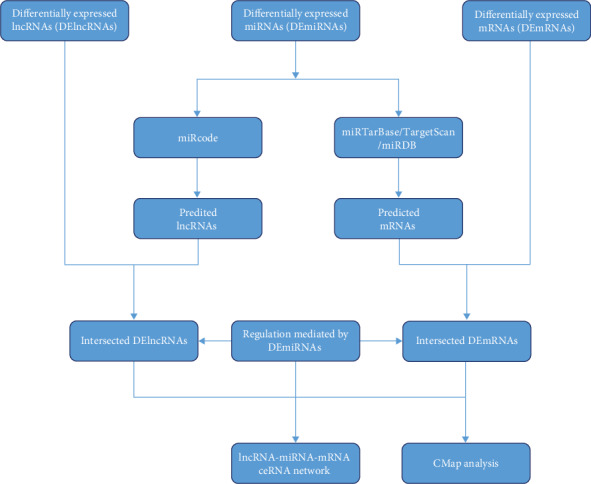
Flowchart of the lncRNA-miRNA-mRNA ceRNA network analysis.

The prediction of miRNA-mRNA interactions was performed on the open-source platform Encyclopedia of RNA Interactomes (ENCORI, http://starbase.sysu.edu.cn) [36]. The unique algorithm of ENCORI enables all obtained interactions to be confirmed by at least one other major RNA-RNA prediction website, such as miRanda, PicTar, or TargetScan. In addition to sequence matching, the prediction was approved by multidimensional sequencing data. All these features make ENCORI a reliable source for predicting RNA-RNA interaction, especially the miRNA-mRNA interaction. Two other databases, miRcode (http://www.mircode.org) and DIANA (http://carolina.imis.athena-innovation.gr), were applied in the study for predicting miRNA-lncRNA interactions. Afterwards, all interactions were input into Cytoscape (version 3.7.2, http://cytoscape.org) to visualize ceRNA regulatory networks. The flow chart can be seen in Figure 1.

### 2.5. Protein-Protein Interaction (PPI) Network

All DEGs were imported into STRING 10.5, which is a search tool used to identify gene interactions (https://string-db.org/). The PPIs were used to construct a network, which was visualized by using Cytoscape software 3.6 (http://www.cytoscape.org). The color of edges in the network indicate protein-protein associations: light blue and purple indicate known interactions from curated database and experimentally determined, respectively; dark green/red/dark blue indicate predicted interactions by gene neighborhood/gene fusions/gene cooccurrence, respectively; and light green/black/blue indicate text mining/coexpression/protein homology.

### 2.6. Gene Expression Signature Analysis with a Connectivity Map

The DEGs were used to perform gene expression signature analysis with connectivity maps (CMap, clue.io). The upregulated and downregulated genes were used as tags, changed into probe IDs referred to Affymetrix U133 GeneChip and uploaded into the CMap database to calculate their values from other drug-target datasets. According to the similarity of gene expression profiles, pairs of gene expression signatures and targeted drugs were used to obtain a value. If the value was a positive number, the target drug would have an effect that was similar to that of the MMD-induced gene expression signature. If the value was a negative number, the targeted drug would have an effect that was opposite that of the MMD-induced gene expression signature; namely, the targeted drug might have an effect that could be useful in treatment.

### 2.7. Statistical Analysis

We used SPSS 11.0 (SPSS, Chicago, IL) to analyze the dataset from the microarray experiments. All data are represented as the mean ± SD. Statistical significance was determined at *P* < 0.05.

## 3. Result and Discussion

### 3.1. Differentially Expressed mRNAs, miRNAs, and lncRNAs between MMD Patients and Healthy Controls

**Figure 2:**
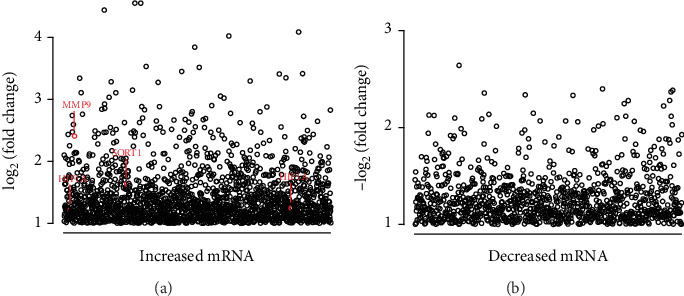
Differential mRNA expression between MMD patients and controls. Log_2_FC > 1 (*P* < 0.05) (MMP9, SORT1, and HIF1*α* are marked in red). *X*-axis shows that the probes of mRNA are arranged in sequence.

After differential expression analysis, a total of 2294 DEmRNAs were screened between MMD patients and healthy controls, 865 of which were downregulated and 1429 of which were upregulated in MMD patients. (Table S1, Figure 2(a)). Several genes reported in previous studies in MMD, such as HIF1*α* (log_2_FC = 1.214), SORT1 (log_2_FC = 1.628), and MMP9 (log_2_FC = 2.40), are marked in Figure 2. HIF1*α* was found to be overexpressed in the intima of the MCA of MMD patients. HIF1*α* is a master transcriptional regulator of the adaptive response to hypoxia. Under hypoxic conditions, HIF1*α* translocates to the nucleus, where the HIF1 complex (HIF*α*/HIF*β*) binds to the hypoxia-response element and activates the expression of many genes that can increase oxygen delivery and respond to oxygen deprivation in MMD [7]. MMP9 belongs to a family of zinc-binding proteolytic enzymes that are capable of degrading all the components of the extracellular matrix in a variety of physiologic and pathophysiological conditions. Fujimura et al. inferred that the higher expression of MMP9 in MMD patients may play an integrated role in physiologic and pathologic angiogenesis and to the instability of the cerebral vascular structure [39]. SORT1 is another gene reported to be associated with MMD. Increased expression of SORT1 inhibited endothelial cell tube formation and regulated major angiogenic factors and MMP9 expression, implying that SORT1 participated in the pathogenesis of MMD [28].

In addition, 94 DEmiRNAs and 3649 DElncRNAs from GEO datasets were identified. Representative DERs are shown in Figure S1 (a-d).

### 3.2. Construction of a Competing Endogenous RNA Regulatory Network

**Figure 3:**
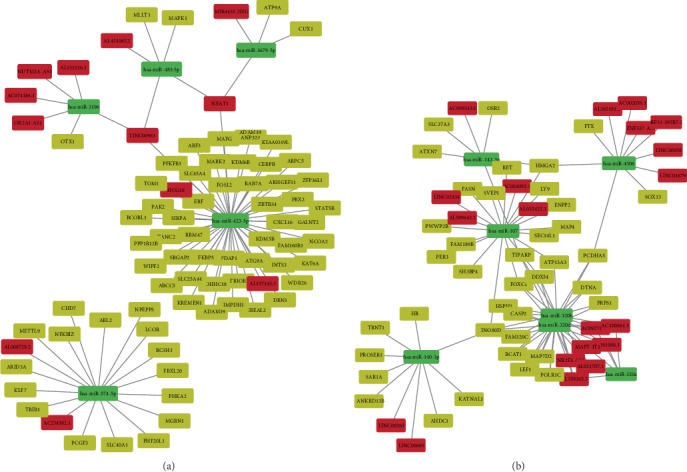
The lncRNA-miRNA-mRNA ceRNA network in MMD. (a) ceRNA network based on upregulated mRNAs involved ceRNA. (b). ceRNA network based on downregulated mRNAs involved ceRNA. Notes: red rectangles represent DElncRNAs, green rectangles represent DEmiRNAs, and yellow rectangles represent DEmRNAs.

The ENCORI database was employed to screen potential interactions between DERs. A synergistic, competitive module of the ceRNA network was constructed separately according to upregulated or downregulated DEmRNAs, which contained 84 nodes in the upregulated group and 66 nodes in the downregulated group. In addition, there were 68 mRNA-miRNA interactions and 16 lncRNA-miRNA interactions in the upregulated group (Figure 3(a)). In the downregulated group, there were 61 interactions between mRNAs and miRNAs and 35 interactions between lncRNAs and miRNAs (Figure 3(b)). The ceRNA network was generated using Cytoscape, as previously discussed.

Based on the network organization, we found that miR-107 competed with 16 mRNAs and 4 lncRNAs (LINC02434, AL589642.1, AC003092.1, and AL035425.3) in the module (Figure 3(b)). A previous study showed that miR-107 is upregulated in response to low-oxygen conditions [40]. Subsequently, miR-107 was found to be abnormally expressed in several cancers, such as PDAC. When miR-107 expression was downregulated in PDAC, cell migration and invasion were inhibited, implying the important role of miR-107 in tumor cell activity [41]. Furthermore, they found that the expression of caveolin-1 was upregulated by a miR-107 inhibitor. Caveolin-1 was reported to be associated with negative remodeling in MMD through the inhibition of angiogenesis in endothelial cells and the induction of apoptosis in VSMCs [30, 31]. Another study by Meng et al. found that miR-107 can inhibit endothelial progenitor cell (EPC) differentiation via HIF1*β* [42]. HIF1*β* is another subunit of HIF1 that generally heterodimerizes with HIF1*α*. Together, they play key roles during hypoxic conditions, which are similar to the conditions in MMD: low oxygen because of vascular occlusion. EPCs can differentiate into mature endothelial cells and play important roles in the recovery of endothelial function and tissue repair. The role of EPCs reflects the mixed state of vascular obstruction and abnormal angiogenesis in the pathogenesis of MMD [43]. The ceRNA network near miR-107 reveals that FoxC1 is one of the potential downstream target genes, and it is necessary in the process of vascular development, involving arterial specification and lymphatic sprouting. Abnormal expression of FoxC1 leads to unusual angiogenesis in many tissues [44, 45].

In addition, miR-423-5p competed with 36 mRNAs (CXCL16, FOSL2, etc.) and 4 lncRNAs (NEAT1, HCG18, AL137145.2, and LINC00963) in the module (Figure 3(a)). miR-423-5p was reported to play important roles in the inhibition of the cell proliferation and invasion of cancer cells such as colon cancer and ovarian carcinoma [46, 47]. Therefore, the downregulation of miR-423-5p in MMD patients may increase the proliferation of vascular smooth muscle cells, which is one likely reason for vessel occlusion. In addition, numerous studies focusing on NEAT1's role in cancer biology found that this lncRNA plays a crucial role in carcinogenesis [48]. NEAT1 mainly works as a ceRNA by sponging antitumor miRNAs [49]. NEAT1 is also involved in immune system responses, viral diseases, and neurodegeneration disorders [50]. To study FOSL2, also named Fra 2, Maurer et al. created Fra 2 knockout mice and found that the mice developed pulmonary arterial occlusion due to vascular SMC proliferation and inflammation and pulmonary fibrosis [51, 52]. All of the above results imply that the ceRNA lncRNA-miRNA-mRNA regulatory network we constructed provides many new clues regarding MMD pathogenesis.

### 3.3. Functional Annotation of the mRNAs Involved in the ceRNA Network

After the ceRNA network was established with the help of the DAVID database, functional annotation and pathway analysis of this small group of DEmRNAs were performed to identify potential candidate pathways or biological processes related to MMD.

**Figure 4:**
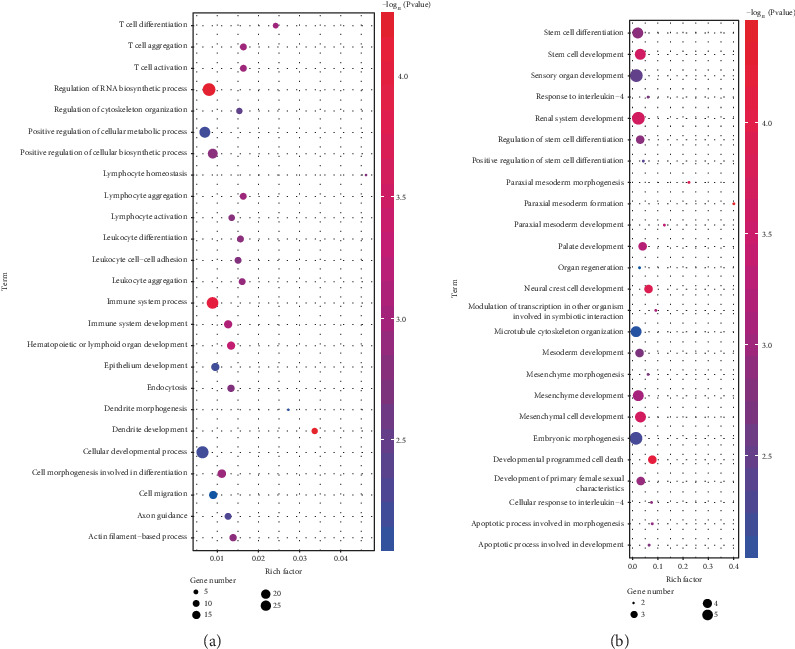
Enriched gene ontology terms for biological process based on the DEmRNAs involved in the ceRNA network.

As shown in Figure 4, some of pathways require our attention, and processes related to the immune response and inflammatory reaction, including immune system process, T cell aggregation, T cell activation, lymphocyte aggregation, and lymphocyte activation, were significantly enriched. Additionally, another enrichment also occurred in biological processes associated with cell development and differentiation, including paraxial mesoderm development and mesenchymal cell differentiation; these results suggest important roles for these biological activities in MMD. The Kyoto Encyclopedia of Genes and Genomes showed that DEmRNAs were enriched in chemokine signaling, ErbB signaling, axon guidance, and vascular smooth muscle contraction (Table 1).

Recently, many studies have shown that immunological/inflammatory factors are involved in the occurrence and development of MMD. According to IHC staining, there were T cells and macrophages infiltrating in the stenosed and thickened vascular intima of MMD patients [53]. The abnormal deposition of IgG in the elastic layer of the ICA and MCA suggests that the infiltration of immune cells and the damage to the immune functions are related to MMD [54]. Moreover, the overexpression of inflammatory factors in MMD patients, such as MCP-1, IL-1*β*, and SDF-1*α*, suggests that inflammation may also affect the progression of MMD [55]. Consistently, in this study, several mRNAs that encode critical inflammatory molecules, such as chemokines and cytokines, were dysregulated and were determined to be DEmRNAs in MMD patients. Nevertheless, although varied mRNAs were clearly enriched in terms of GO analysis, there were few found in the ceRNA network. However, several important genes involved in the regulation of inflammation in MMD were modulated by ceRNAs. CXCL16 is considered to be an important pathogenic mediator of atherosclerosis (clinical severity is graded according to the severity of carotid stenosis) [56]. CXCL16 is a vascular-derived factor that induces angiogenesis [57]. CXCL16 also exists in a soluble form and interacts with its specific chemokine receptor, CXCR6, to recruit the migration of activated T cells into the inflammatory tissue [58]. As shown in Figure 3(b), four potential lncRNAs, including LINC00963, NEAT1, HCG18, and AL137145.2, could act as ceRNAs to regulate CXCL16 through miR-107. The work on this interesting ceRNA network remains to be done in the future.

### 3.4. Protein-Protein Interaction (PPI) Network

**Figure 5:**
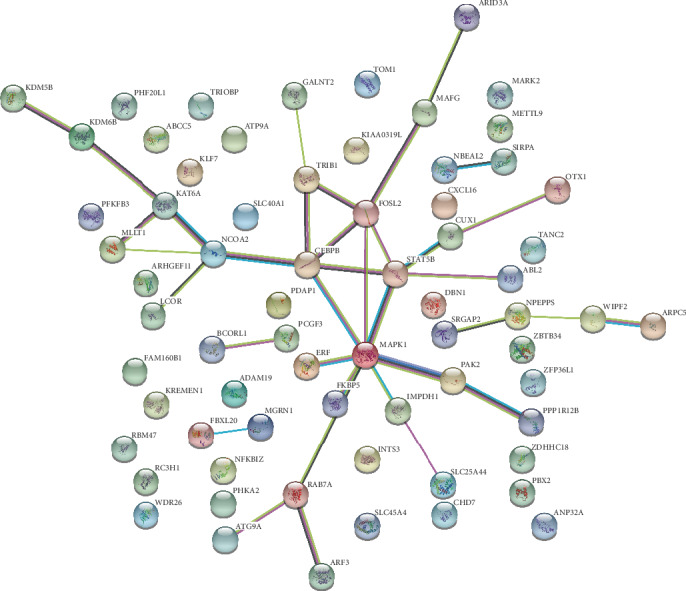
The protein-protein interaction network. The red and green nodes represent the regulated and downregulated genes, respectively. (For interpretation of the references to color in this figure legend, the reader is referred to the online version of this article.)

As shown in Figure 5, a PPI network for DEmRNA-involved ceRNA networks was constructed by Cytoscape software. It is important to highlight that some striking genes, such as MAKP1, STAT5B, CEBPB, FOSL2, PAK2, and ABL2, play vital key roles in MMD. These interesting genes were also shown in Table 1, such as ABL2, PAK2, MAPK1, and STAT5B were enriched in the ErbB signaling pathway. After the identification of the overlap between the above genes, chemokine signaling, T cell receptor signaling, and ErbB signaling shed some light on the pathogenesis of MMD.

### 3.5. Potential Small Molecule Drugs

**Figure 6:**
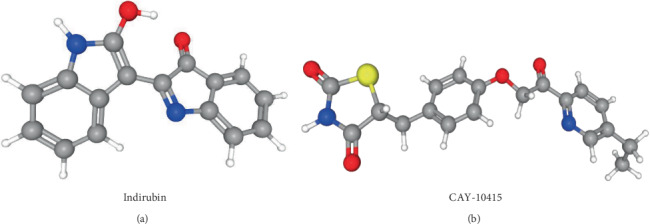
Potential molecular drugs. (a) Indirubin. (b) CAY-10415.

All the DEmRNAs involved in the ceRNA regulatory network in MMD were analyzed by CMap to identify small molecule drugs. Strong negative correlations were found between MMD and enzastaurin, cyproheptadine, flupirtine, indirubin, and mitoglitazone (CAY-10415); strong positive correlations were found between MMD and flavokavain-b, CGS-20625, vinburnine, apicidin, and cytochalasin-d (Table S5). The drugs that had a strong negative correlation with the pathogenesis of MMD might have therapeutic effects on MMD (Table 2). CAY-10415 and indirubin gained our attention. The structures of the two potential molecular drugs were investigated using the PubChem database (Figure 6). CAY-10415 is a member of a new class of compounds that modulate mitochondrial pyruvate carrier (MPC), a key controller of cellular metabolism that influences mTOR activation [59]. It is commonly known that CAY-10415 can be used as an insulin sensitizer, and it can play this role without activating PPARᵧ. Therefore, CAY-10415 can avoid negative side effects observed in currently used insulin sensitizers, such as pioglitazone and rosiglitazone. CAY-10415 has been used in Alzheimer's disease patients [60]. It is generally accepted that insulin sensitizers can not only improve diabetes but also improve blood lipid disorders, reduce the level of free fatty acids in plasma, reduce the effect of fat toxicity, and indirectly protect the function of *β* cells [61]. By inhibiting the proliferation and migration of vascular smooth muscle cells and reducing the intima-media thickness of arteries, it can play a protective role in the intima. Likewise, indirubin, a red isomer of indigo, is the active ingredient of the traditional Chinese drug *Danggui Longhui Wan*, which was used for the treatment of chronic myelocytic leukemia (CML) [62]. Enzyme-based *in vitro* studies have observed that indirubin and its derivatives, such as indirubin-3′-monoxime, indirubin-5-sulfonate, and indirubin-3′-monoxime-5-sulphonic acid, are potential inhibitors of CDKs [63]. Furthermore, different indirubin derivatives showed antiangiogenesis activity by blocking VSMC proliferation and endothelial cell function through the inhibition of the STAT signaling pathway and reduction of neointima formation *in vivo* [64]. All of the above findings suggest that CAY-10415 and indirubin may be used in MMD patients to avoid vascular aberration and occlusion.

## 4. Conclusions

**Figure 7:**
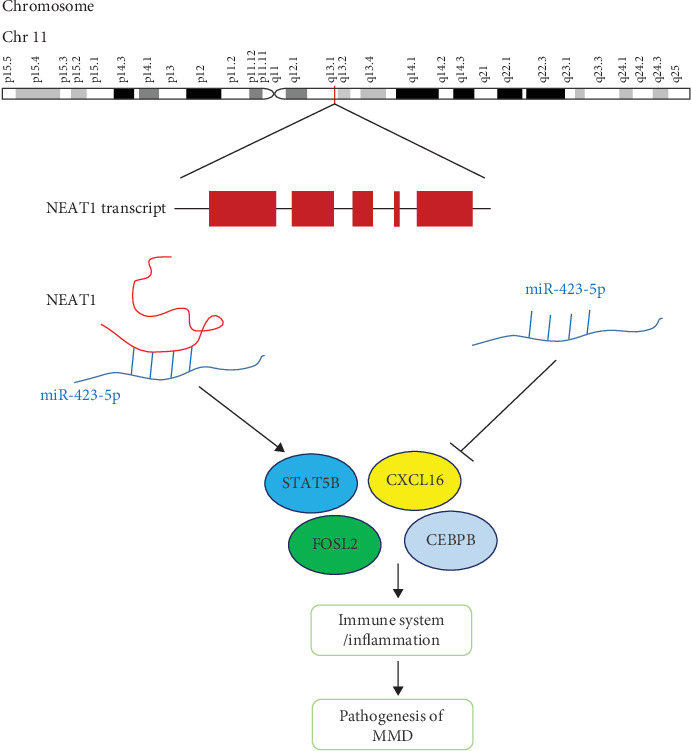
The potential mechanism of a DElncRNA sponging miR-423-5p.

In summary, using bioinformatics analysis of candidate RNAs, the present study identified a series of clearly altered lncRNAs, miRNAs, and mRNAs involved in MMD. Furthermore, a ceRNA lncRNA-miRNA-mRNA regulatory network was constructed, which provides a novel insight into the molecular pathogenesis of MMD, thus giving promising clues for clinical therapy. In addition, core regulatory miRNAs (miR-107 and miR-423-5p) and key mRNAs (STAT5B, FOSL2, CEBPB, and CXCL16) were enriched in immune system/inflammation biological processes, indicating their potential role in MMD (Figure 7). In the future, more attention should be paid to the validation of competing endogenous RNA interactions with experimental techniques. Finally, two potential small molecule drugs, CAY-10415 and indirubin, were identified by CMap to be candidate drugs for treating MMD.

## Figures and Tables

**Table:** (a) KEGG pathway enrichment of decreased mRNA involved in the ceRNA network

KEGG ID	KEGG term	*P* value	Symbols
05216	Thyroid cancer	0.0012614	RET, LEF1
00230	Purine metabolism	0.0353649	PRPS1, POLR1C
04141	Protein processing in the endoplasmic reticulum	0.0365787	HSPH1, SAR1A

**Table:** (b) KEGG pathway enrichment of increased mRNA involved in the ceRNA network

KEGG ID	KEGG term	*P* value	Symbols
04012	ErbB signaling pathway	0.000117	ABL2, PAK2, MAPK1, STAT5B
04270	Vascular smooth muscle contraction	0.004943	PPP1R12B, MAPK1, ARHGEF11
04380	Osteoclast differentiation	0.006506	FOSL2, MAPK1, SIRPA
04360	Axon guidance	0.006648	PAK2, MAPK1, SRGAP2
05221	Acute myeloid leukemia	0.012833	MAPK1, STAT5B
05131	Shigellosis	0.014609	MAPK1, ARPC5
04062	Chemokine signaling pathway	0.018775	MAPK1, STAT5B, CXCL16
05211	Renal cell carcinoma	0.018968	PAK2, MAPK1
05220	Chronic myeloid leukemia	0.020529	MAPK1, STAT5B
04810	Regulation of actin cytoskeleton	0.025711	PAK2, MAPK1, ARPC5
04666	Fc gamma R-mediated phagocytosis	0.032876	MAPK1, ARPC5
04660	T cell receptor signaling pathway	0.042378	PAK2, MAPK1

**Table 2:** List of the top 10 potential small molecular drugs predicted by CMap. Scores indicate the strong negative correlation found between MMD and drugs.

	Score	Type	ID	Name	Description
1	-88.5	cp	BRD-K86727142	Embelin	HCV inhibitor
2	-88.74	cp	BRD-K11630072	Carmofur	Thymidylate synthase inhibitor
3	-90.86	cp	BRD-A61858259	CAY-10415	Insulin sensitizer
4	-91.54	cp	BRD-K04923131	GSK-3-inhibitor-IX	Glycogen synthase kinase inhibitor
5	-91.85	cp	BRD-K53959060	Indirubin	CDK inhibitor
6	-93.79	cp	BRD-K50720187	Flupirtine	Glutamate receptor antagonist
7	-93.87	cp	BRD-A14985772	Ascorbyl-palmitate	Antioxidant
8	-94.57	cp	BRD-K28143534	Cyproheptadine	Histamine receptor antagonist
9	-95.78	cp	BRD-K79404599	Enzastaurin	PKC inhibitor
10	-97.25	cp	BRD-K89687904	PKCbeta-inhibitor	PKC inhibitor

## Data Availability

(1) The miRNA microarray data used to support the findings of this study have been deposited in the Gene Expression Omnibus (GEO, https://www.ncbi.nlm.nih.gov/geo/) in NCBI (GSE45737). (2) The lncRNA and mRNA microarray data included in this study are available upon request by contact with the corresponding author. The data were kindly provided by a collaborating Prof. Zhao.

## Supplementary Materials

Supplementary Materials Table S1: DEmRNA list in excel file. Table S2: DElncRNA list in excel file. Table S3: increased miRNA list in excel file. Table S4: decreased miRNA list in excel file. Table S5: drugs in excel file. Figure S1: DElncRNA and DemiRNA in tiff format.
