# Supplementary figures and images for: Construction and Comprehensive Analysis of Dysregulated Long Noncoding RNA-Associated Competing Endogenous RNA Network in Moyamoya Disease
Source: Comput Math Methods Med. 2020 Jun 13;2020:2018214. doi: 10.1155/2020/2018214 (PMC7306867; doi:10.1155/2020/2018214)

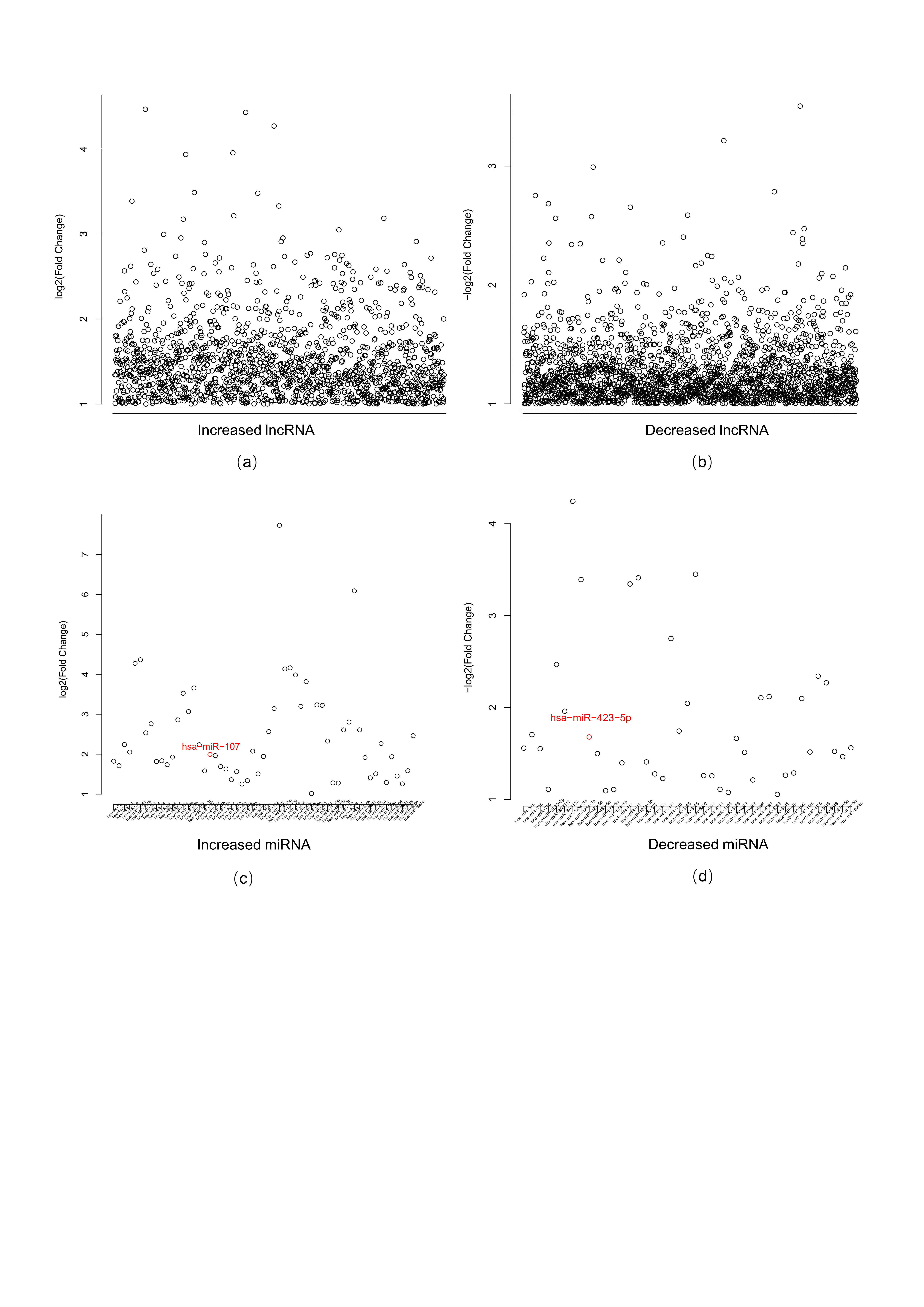

Supplement: Supplementary Materials — Table S1: DEmRNA list in excel file. Table S2: DElncRNA list in excel file. Table S3: increased miRNA list in excel file. Table S4: decreased miRNA list in excel file. Table S5: drugs in excel file. Figure S1: DElncRNA and DemiRNA in tiff format. [file 2018214.f1.zip › Figure S1.tif]
